# Data from identification of diagnostic biomarkers and metabolic pathway shifts of heat-stressed lactating dairy cows

**DOI:** 10.1016/j.dib.2015.04.020

**Published:** 2015-05-07

**Authors:** He Tian, Weiyu Wang, Nan Zheng, Jianbo Cheng, Songli Li, Yangdong Zhang, Jiaqi Wang

**Affiliations:** a Institute of Animal Science, Chinese Academy of Agricultural Sciences, Beijing, China; b The High School Affiliated to Renmin University of China, Beijing, China; c College of Animal Science and Technology, Anhui Agricultural University, Hefei, China

**Keywords:** Metabolomics, Lipidomics, Heat stress, Lactating dairy cows

## Abstract

Controlling heat stress (HS) is a global challenge for the dairy industry. In this work, an integrated metabolomics and lipidomics approach using ^1^H nuclear magnetic resonance (NMR) and ultra-fast LC–MS in combination with multivariate analyses was employed to investigate the discrimination of plasma metabolic profiles between HS-free and HS lactating dairy cows. Here we provide the information about the acquiring and processing of raw data obtained by ^1^H NMR and LC–MS techniques. The data of present study are related to the research article “Identification of diagnostic biomarkers and metabolic pathway shifts of heat-stressed lactating dairy cows” in the Journal of Proteomics (Tian et al., J. Proteomics, (2015), doi:10.1016/j.jprot.2015.04.014).

Specifications table

**Table:** 

Subject area	Biology
More specific subject area	Plasma metabolomics
Type of data	Table, figure, excel file
How data was acquired	^1^H NMR and UFLC–MS (Bruker Biospin Billerica)
	Ultra-fast LC(Shimadzu)
	5600 Triple TOF mass spectrometer(Applied Biosystems/MDS Sciex)
	QTRAP 5500 (Applied Biosystems/MDS Sciex)
	TopSpin software (version 3.0; Bruker Biospin)
	AMIX software package (version 3.8.3, Bruker Biospin)
	MATLAB R2012a software (MathWorks, Natick, MA, USA)
	SIMCA-P 12.0 software package (Umetrics AB, Umeå, Sweden)
	Analyst^®^ TF 1.6 Software (Applied Biosystems/MDS Sciex)
	open-source XCMS package (version 1.20.1)
	Human Metabolome database and METLIN database
Data format	Analyzed
Experimental factors	Plasma samples from Chinese Holstein cows with and without heat stress were collected to characterize the metabolic changes induced by heat stress (HS)
Experimental features	Integrated metabolomics and lipidomics using ^1^H nuclear magnetic resonance (NMR) and UFLC–MS techniques
Data source location	Beijing, China
Data accessibility	Programs of data transformation are directly provided with this article

Value of the data

The data of multivariate analysis highlight the significant differences of plasma metabolic profiling between HS and HS-free dairy cows.

The data point out the potential biomarkers of HS dairy cows.

The data provide insights into shifted pathways of dairy cows induced by HS.

## 1 Data, experimental design, materials and methods

### 1.1 Sample collection and experimental design

**Fig. 1:**
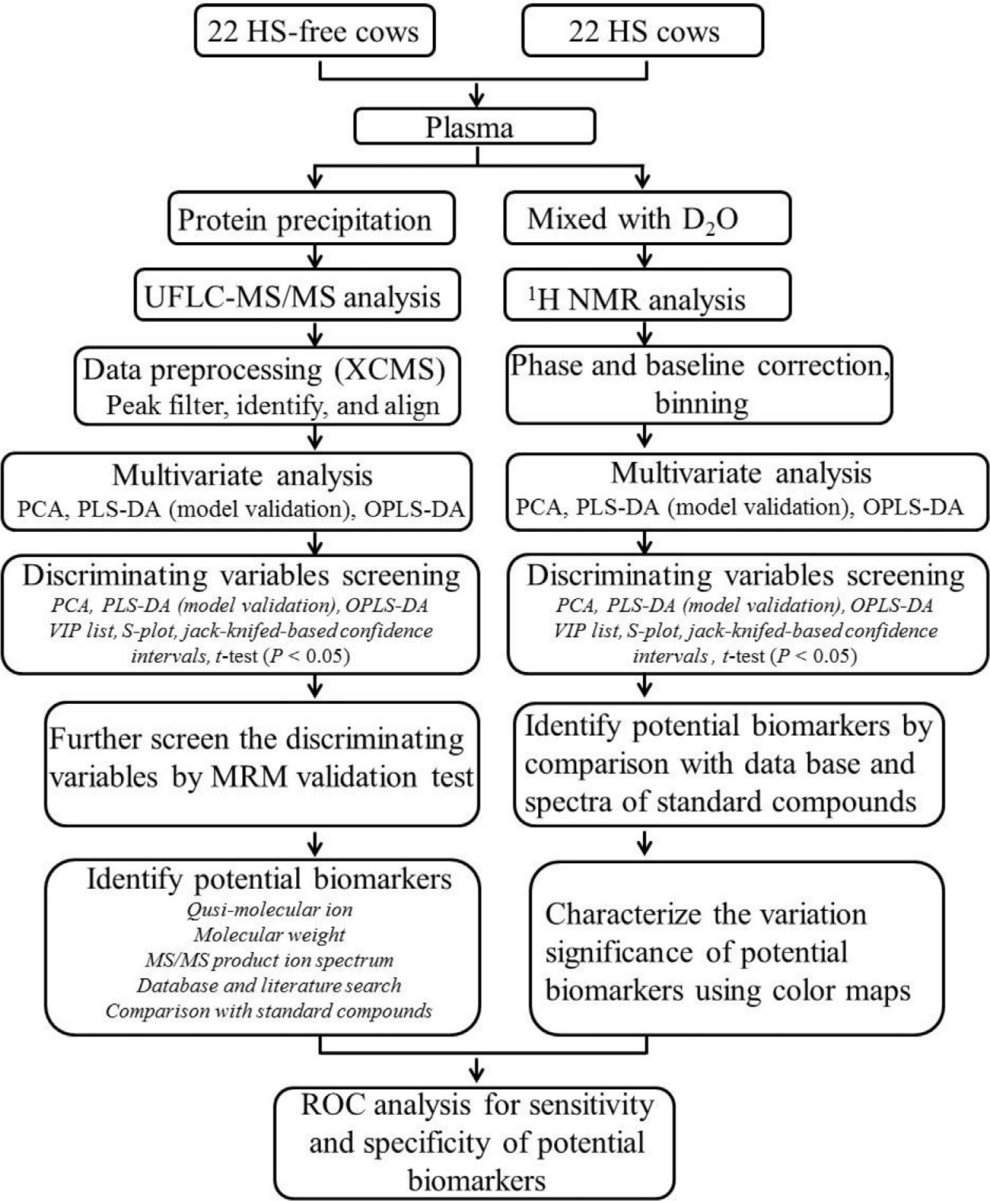
**Overview of the strategy used to identify diagnostic biomarkers of HS in lactating dairy cows.** HS—heat stress; NMR—nuclear magnetic resonance; MVDA—multivariate statistical data analysis.

All experiments involving animals were conducted according to the principles of the Chinese Academy of Agricultural Sciences Animal Care and Use Committee (Beijing, China). Fasting blood samples were collected before morning feeding from the caudal veins of Holstein dairy cows, put into K2 EDTA anti-coagulation vacuum tubes, and centrifuged at 1600*g* for 10 min at 4 °C. The supernatants were transferred to tubes, frozen quickly, and stored at −80 °C until use. An overview of the experimental design to acquire the data in present article is shown Fig. 1.

### 1.2 Sample preparation

For LC–MS metabolomics analysis, 150 μL aliquots of each thawed plasma sample were mixed with 600 μL ice-cold acetonitrile, vortexed, and centrifuged for 10 min at 10,000 rpm and 4 °C. The 650 μL supernatant of each sample was transferred to another tube, and concentrated to dryness with a SpeedVac Concentrator (SPD121P, Thermo Savant, Waltham, MA, USA). Each dried sample was reconstituted in 100 μL ACN/H_2_O (1:99 v/v), and filtered through a Captiva 96-well filter plate (Agilent, Santa Clara, CA, USA) for analysis.

For analyses of the plasma lipidome, a modified preparation method was used [2]. Briefly, a 30 µL aliquot of each plasma sample was mixed with 200 µL methanol, followed by the addition of 660 µL methyl *tert*-butyl ether, and vortexing of the sample. Subsequently, 150 µL water were added to each tube, the samples were vortexed for 5 min, incubated for 5 min, and centrifuged for 5 min at 10,000 rpm and 4 °C. The upper layers (500 µL) were collected, evaporated to dryness using a SpeedVac SPD121P concentrator, and re-dissolved in 500 µL of ACN/isopropanol/H_2_O (65:30:5 v/v/v). The corresponding solvents were filtered through a Captiva 96-well filter plate for LC–MS analysis.

For NMR analysis, the plasma samples were centrifuged for 10 min at 1600*g* and 4 °C. The supernatants (20 μL) were collected, mixed with 40 μL of deuterium oxide containing 1 mM 3-(trimethylsilyl) propionic-2,2,3,3,d4 acid sodium salt, and transferred to 1.7 mm NMR tubes for analysis.

### 1.3 NMR spectroscopic analysis

^1^H NMR spectra were acquired using an automated NMR Case sample changer on a Bruker AvanceIII 500 spectrometer (Bruker Biospin Billerica, MA, USA) operating at 500.13 MHz and equipped with a 1.7-mm triple resonance inverse probe at 298 K. A Carr–Purcell–Meiboom–Gill pulse sequence was used to enhance the contribution of low-molecular weight metabolites, and a diffusion-edited experiment with bipolar pulse pair-longitudinal eddy current delay pulse sequence was used to measure the lipid content of plasma lipoproteins [3]. The Carr–Purcell–Meiboom–Gill method utilized a spin–spin relaxation delay (2*nτ*) of 320 ms for each sample, with water signal irradiation applied during the recycle delay. For bipolar pulse pair-longitudinal eddy current delay, a sine shaped gradient with a strength of 32 G/cm and a duration of 2.5 ms was followed by a delay of 200 μs to allow for the decay of eddy currents. The diffusion delay was 120 ms and the time delay (*T*_*e*_) was 5 ms. A line-broadening factor of 1 Hz was applied to free induction decays before Fourier transformation. Spectra were acquired with 128 scans and then zero-filled and Fourier-transformed to 128k data points. Representative NMR spectra plasma samples are shown in Supplementary Fig. 1A.

Metabolites were identified by inserting the experimental spectra into the Chenomx spectral database (Edmonton, AB, Canada), and comparing them with the spectra of standard compounds.

The significance of variation in the metabolic profiling data between the HS-free group and corresponding HS group was determined using SIMCA-P to generate metabolite correlations between groups (see correlation results in Supplementary Excel); then, MATLAB R2012a software was applied for the color map visualization (see Supplementary running program 1).

### 1.4 LC–MS/MS analyses

LC–MS/MS analyses were performed using Shimadzu ultra-fast LC (UFLC) 20ADXR system and a 5600 Triple TOF mass spectrometer (Applied Biosystems/MDS Sciex, Concord, ON, Canada).

Metabolome analyses utilized ACQUITY UPLC HSS T_3_ 1.8 µm, 2.1×100 mm columns (Waters, Dublin, Ireland). The temperatures of the column and auto-sampler were maintained at 40 °C and 4 °C, respectively. The injection volume was 5 µL per run, and the flow-rate was 0.3 mL/min. Mobile phases A and B were 0.1% formic acid in de-ionized water and ACN, respectively. The linear gradients were: 1% B at 1 min, 40% B at 5 min, 50% B at 8 min, 65% B at 10 min, 76% B at 16 min, 100% B at 25 min, 1% B at 25.1 min, and 1% B at 30 min.

The ESI source was set up in positive and negative ionization modes. The MS parameters for detection were: ESI source voltage 5.5 kV or −4.5 kV; vaporizer temperature, 550 °C; drying gas (N_2_) pressure, 60 psi; nebulizer gas (N_2_) pressure, 60 psi; curtain gas (N_2_) pressure, 30 psi; and declustering potential, 50 V. The scan range was *m/z* 60–1000. Data acquisition and processing were performed using Analyst^®^ TF 1.6 Software (Applied Biosystems/MDS Sciex).

Lipidome analysis used ACQUITY CSHTM C18 1.7 µm, 2.1×100 mm columns (Waters, USA). The mobile phase consisted of 0.1% formic acid and 10 mM ammonium formate in de-ionized water (mobile phase A) or ACN: isopropanol (1:1; mobile phase B). The auto-sampler temperature was maintained at 10 °C. The linear gradients were: 70% B at 1 min, 99% B at 15 min, 99% B at 20 min, 1% B at 20.1 min, and 1% B at 25 min. The MS was set up in positive ionization mode. The scan range was *m/z* 200–1000. Representative total ion chromatograms of plasma samples are shown in Supplementary Fig. 1B–D.

Information-dependent acquisition mode was used for MS/MS analyses of the metabolome and lipidome. The collision energy was 35 eV.

High-resolution MS, isotope abundance ratios, MS/MS, the Human Metabolome database, the METLIN database, a literature search, and standard comparisons were employed to identify ion structures.

Quality control (QC) samples were detected once every 10 LC–MS runs to monitor the reproducibility of the instrument. Plasma samples from different groups were randomly alternated during the analysis. Auto-calibration was performed using the Calibrant Delivery System in both positive and negative ion modes.

### 1.5 Data handling

Raw LC–MS data files (.wiff) were converted into mzXML format using ProteoWizard (http://metlin.scripps.edu/xcms/download/pwiz/pwiz.zip). The files were processed using an open-source XCMS package (version 1.20.1) in R statistical software (version 2.10.0) for peak discrimination, filtering and alignment [4], see the Supplementary running program 2. During XCMS implementation, the R-package CAMERA (Collection of Algorithms for Metabolite Profile Annotation) was used to annotate the isotope, adduct, and product ion peaks. In combination with the corresponding extracted ion chromatograms, these ions were manually excluded from the acquired peaks. The resulting two-dimensional matrices, including the observations (sample names) in columns, the variables (*m/z*-retention time pairs) in rows, and the peak areas, were imported into the SIMCA-P 12.0 software package (Umetrics AB, Umeå, Sweden) for multivariate analysis.

The raw ^1^H NMR spectra were manually corrected for phase and baseline distortions using TopSpin software (version 3.0; Bruker Biospin) and were referenced to the signal of 3-(trimethylsilyl) propionic-2,2,3,3,d4 acid sodium salt (δ 0.0 ppm). The ^1^H NMR spectra of plasma specimens were binned into 0.01 ppm integral regions and integrated in the 0.5–6.0 ppm region using the AMIX software package (version 3.8.3, Bruker Biospin). The regions containing the water resonance (δ 5.12–4.7) were removed. The spectra were normalized to the total sum of the spectral integrals to compensate for differences in sample concentration. Multivariate analyses of the normalized NMR data sets were performed using the SIMCA-P 12.0 software package.

Prior to multivariate analysis, pareto and center scaling were respectively applied to LC–MS and ^1^H NMR data to reduce noise and artifacts in the models. Data from Quality Control samples were analyzed by Principal component analysis (PCA) to monitor the reproducibility of the instrument (see Supplementary Fig. 2), based on the judgment of whether peak areas deviation are less than two standard deviations [5,6]. The retention time deviation profiles of LC–MS data that were generated using an open-source XCMS package were analyzed to evaluate the reproducibility of the chromatography (see Supplementary Fig. 3), based on the judgment of whether deviations are less than 20 s for most analyses [6]. PCA was performed to visualize global clustering and separation trends, or outliers (see Supplementary Fig. 4). Partial least squares dicriminant analysis (PLS-DA) models were applied to validate the model against overfitting through 999 random permutation tests (see Supplementary Fig. 5). The models of orthogonal partial least squares discriminate analysis (OPLS-DA) on the metabolic profiles of HS-free and HS groups were constructed (see Supplementary Fig. 6). Discriminating variables were selected according to the *S*-plots (see Supplementary Fig. 7), jack-knifed-based confidence intervals (see Supplementary Fig. 8), variable importance in projection values (VIP>1), and raw data plots in the OPLS-DA models (examples of Supplementary Fig. 9) [6–8]. Furthermore, an independent *t*-tests (*P*<0.05) (SPSS version 13.0) were used to determine if the differences between the concentrations of candidate biomarkers obtained from OPLS-DA of the HS-free and HS groups were statistically significant.

### 1.6 Ultra-fast LC–MS/MS-based verification test

The conditions of chromatographic gradient elution were unchanged. The eluent was injected into a triple quadrupole-trap mass spectrometer equipped with an ESI source (QTRAP 5500, Applied Biosystems/MDS Sciex). All of the LC–MS-discovered potential candidates were detected by ultra-fast LC–MS/MS in multiple reaction monitoring (MRM) mode. For each analyte of interest, the collision energies and precursor/fragment ion pairs were pre-optimized to generate an optimal signal to noise ratio. The MRM transitions were listed in Supplementary Tables 1 and 2. The internal standards levofloxacin (362→261) and hesperidin (611→303) were used for detecting the metabolome and lipidome candidate ions in positive ion mode; and the internal standards rhein (293→221) and hesperidin (609→301) were included for detecting the metabolome candidate ions in negative ion mode.

### 1.7 Characterization of potential diagnostic biomarkers

The sensitivity and specificity of all candidates were evaluated by plotting ROC curves using SPSS (version 13.0), and calculating the area under the curves (AUC) [6]. The discriminatory power of biomarker candidates was ranked and visualized by heat maps (see Supplementary Fig. 10).

## Author declaration

We wish to confirm that there are no known conflicts of interest associated with this publication and there has been no significant financial support for this work that could have influenced its outcome.

We confirm that the manuscript has been read and approved by all named authors and that there are no other persons who satisfied the criteria for authorship but are not listed. We further confirm that the order of authors listed in the manuscript has been approved by all of us.

We confirm that we have given due consideration to the protection of intellectual property associated with this work and that there are no impediments to publication, including the timing of publication, with respect to intellectual property. In so doing we confirm that we have followed the regulations of our institutions concerning intellectual property.

We further confirm that any aspect of the work covered in this manuscript that has involved experimental animals has been conducted with the ethical approval of the principles of the Chinese Academy of Agricultural Sciences Animal Care and Use Committee (Beijing, China), and that such approvals are acknowledged within the manuscript.

We understand that the Corresponding Author is the sole contact for the Editorial process (including Editorial Manager and direct communications with the office). He/she is responsible for communicating with the other authors about progress, submissions of revisions and final approval of proofs. We confirm that we have provided a current, correct email address which is accessible by the Corresponding Author and which has been configured to accept email from wang-jia-qi@263.net.

He Tian, April 25, 2015.

Weiyu Wang, April 25, 2015.

Nan Zheng, April 25, 2015.

Jianbo Cheng, April 25, 2015.

Songli Li, April 25, 2015.

Yangdong Zhang, April 25, 2015.

Jiaqi Wang, April 25, 2015.
