# Supplementary material for: Data from identification of diagnostic biomarkers and metabolic pathway shifts of heat-stressed lactating dairy cows
Source: Data Brief. 2015 May 7;4:90–5. doi: 10.1016/j.dib.2015.04.020 (PMC4510399; doi:10.1016/j.dib.2015.04.020)
Supplement: Supplementary file 1 — Supplementary material [file mmc1.zip › Supplementary file 3.docx]

**Data in Brief**

**Data from identification of diagnostic biomarkers and metabolic pathway shifts of heat-stressed lactating dairy cows**

He Tian, Weiyu Wang, Nan Zheng, Jianbo Cheng, Songli Li, Yangdong Zhang, Jiaqi Wang

**Program 1. Application for color map visualization of the significance of variation in the metabolic profilings between the HS-free and corresponding HS groups using MATLAB R2012a software**

data = xlsread('Book1.xls');

X = data(:,1);

Y = data(:,5);

C = data(:,6);

color_line(X,Y,C,'LineWidth',1.5);

set(gca, 'XDir','reverse');

axis([0.5,9,-2E4,2.2E4])

xlabel('Chemical Shift (ppm)','FontWeight','Demi');

ylabel('Coefficients', 'FontWeight','Demi');

caxis([0 1.0])

t1=linspace(0,1,6);

colorbar('Ylim',[0,1.0],'ytick',t1);

set(gca, 'LineWidth',2);

set(gca, 'FontWeight','Demi');

box;

**Program 2. Application for peak discrimination, filtering, alignment and CAMERA analysis in UFLC-MS data acquired at positive ionization mode.**

rm(list=ls(all=TRUE)）

library(Biobase)

library(xcms)

library(multtest)

library(CAMERA)

sessionInfo()

xs<-xcmsSet(profmethod = "binlin",method="centWave",ppm = 20, peakwidth=c(5,20), snthresh =20, prefilter=c(3,100),mzdiff =0.05)

xs

xs<-group(xs,bw=10,minfrac=0.5)

save(xs,file="xs.Rda")

ret.xs.obiwarp <-retcor(xs,method="obiwarp",plottype="deviation")

ret.xs.obiwarp<-group(ret.xs.obiwarp, bw=10,minfrac=0.5)

ret.xs.obiwarp

save(ret.xs.obiwarp, file="ret.xs.obiwarp.Rda")

fill.ret.xs.obiwarp<-fillPeaks(ret.xs.obiwarp)

fill.ret.xs.obiwarp

save(fill.ret.xs.obiwarp, file="fill.ret.xs.obiwarp.Rda")

an.A<-annotate(fill.ret.xs.obiwarp,sigma=6,perfwhm=0.3,cor_eic_th=0.75,maxcharge=3,maxiso=3,mzabs=0.03,multiplier=3,polarity="positive")

peaklist.A<-getPeaklist(an.A)

write.csv(peaklist.C,file='annotated.A.csv')
