# Supplementary material for: Data from identification of diagnostic biomarkers and metabolic pathway shifts of heat-stressed lactating dairy cows
Source: Data Brief. 2015 May 7;4:90–5. doi: 10.1016/j.dib.2015.04.020 (PMC4510399; doi:10.1016/j.dib.2015.04.020)
Supplement: Supplementary file 1 — Supplementary material [file mmc1.zip › Supplementary file 1.doc]

**Data in Brief**

**Data from identification of diagnostic biomarkers and metabolic pathway shifts of heat-stressed lactating dairy cows**

He Tian, Weiyu Wang, Nan Zheng, Jianbo Cheng, Songli Li, Yangdong Zhang, Jiaqi Wang

**Table 1 -** Transition list of metabolome candidate ions verified by LC-MS/MS in MRM mode.

| Ionization mode | RT (min) | Precursor ion (Q1) | Product ion (Q3) | Ionization mode | RT (min) | Precursor ion (Q1) | Product ion (Q3) |
| --- | --- | --- | --- | --- | --- | --- | --- |
| [M + H]^+^ | 0.94 | 118.1 | 58.1 | [M + H]^+^ | 15.30 | 482.3 | 184.1 |
| [M + H]^+^ | 1.32 | 121.1 | 61.1 | [M + H]^+^ | 12.39 | 496.3 | 184.1 |
| [M + H]^+^ | 1.86 | 136.1 | 109.1 | [M + H]^+^ | 15.91 | 510.3 | 184.1 |
| [M + H]^+^ | 10.59 | 293.1 | 132.1 | [M + H]^+^ | 12.16 | 520.3 | 184.1 |
| [M + H]^+^ | 1.11 | 317.1 | 213.1 | [M + H]^+^ | 8.04 | 511.3 | 485.2 |
| [2M + Na]^+^ | 0.91 | 383.1 | 203.1 | [M + H]^+^ | 12.01 | 362.3 | 261.2 |
| [M + H]^+^ | 12.95 | 122.1 | 79.1 | [M - H]^-^ | 0.97 | 367.1 | 187.1 |
| [M + H]^+^ | 3.86 | 149.1 | 65.1 | [M - H]^-^ | 9.41 | 453.3 | 407.3 |
| [M + H]^+^ | 4.37 | 159.1 | 117.1 | [M - H]^-^ | 1.81 | 89.1 | 41.1 |
| [M + H]^+^ | 1.14 | 165.1 | 77.1 | [M - H]^-^ | 4.51 | 187.1 | 107.1 |
| [M + H]^+^ | 1.12 | 204.1 | 85.1 | [M - H]^-^ | 13.87 | 476.3 | 279.2 |
| [M + H]^+^ | 5.29 | 180.1 | 77.1 | [M - H]^-^ | 11.07 | 407.3 | 343.3 |
| [M + H]^+^ | 0.95 | 176.1 | 70.1 | [M - H]^-^ | 0.93 | 131.1 | 85.1 |
| [M + H]^+^ | 0.96 | 175.1 | 70.1 | [M - H]^-^ | 9.57 | 464.3 | 74.1 |
| [M + H]^+^ | 1.12 | 182.1 | 91.1 | [M - H]^-^ | 13.60 | 526.3 | 241.2 |
| [M + H]^+^ | 1.09 | 116.1 | 70.1 | [M - H]^-^ | 13.74 | 197.8 | 162.8 |
| [M + H]^+^ | 6.15 | 186.1 | 114.1 | [M - H]^-^ | 4.78 | 338.1 | 160.1 |
| [M + H]^+^ | 3.84 | 188.1 | 118.1 | [M - H]^-^ | 6.07 | 187.1 | 160.1 |
| [M + H]^+^ | 1.06 | 132.1 | 90.1 | [M - H]^-^ | 11.80 | 510.3 | 450.3 |
| [M + H]^+^ | 7.69 | 208.1 | 188.1 | [M - H]^-^ | 15.29 | 265.2 | 96.9 |
| [M + H]^+^ | 3.23 | 224.1 | 167.1 | [M - H]^-^ | 9.63 | 448.3 | 74.1 |
| [M + H]^+^ | 5.29 | 194.1 | 91.1 | [M - H]^-^ | 21.29 | 465.3 | 96.9 |
| [M + H]^+^ | 8.81 | 274.3 | 256.3 | [M - H]^-^ | 1.09 | 343.1 | 256.1 |
| [M + H]^+^ | 12.99 | 279.1 | 149.1 | [M - H]^-^ | 1.58 | 264.8 | 206.8 |
| [M + H]^+^ | 8.95 | 318.3 | 256.2 | [M - H]^-^ | 7.02 | 420.1 | 187.1 |
| [M + H]^+^ | 8.79 | 320.2 | 71.1 | [M - H]^-^ | 3.54 | 241.1 | 183.1 |
| [M + H]^+^ | 0.91 | 104.1 | 58.1 | [M - H]^-^ | 14.69 | 480.3 | 255.2 |
| [M + H]^+^ | 10.23 | 427.2 | 356.2 | [M - H]^-^ | 19.32 | 303.2 | 259.2 |
| [M + H]^+^ | 9.86 | 450.3 | 414.3 | [M - H]^-^ | 20.81 | 281.2 | 210.2 |
| [M + H]^+^ | 10.31 | 302.3 | 284.3 | [M - H]^-^ | 19.53 | 279.2 | 261.2 |
| [M + H]^+^ | 11.75 | 432.2 | 119.1 | [M - H]^-^ | 4.35 | 283.2 | 211.2 |
| [M + H]^+^ | 11.34 | 494.3 | 184.1 | [M - H]^-^ | 14.85 | 609.5 | 301.3 |
| [M + H]^+^ | 15.01 | 524.3 | 184.1 |  |  |  |  |

**Table 2 -** **Transition list of lipidome candidate ions verified by LC-MS/MS in MRM mode.**

| Ionization mode | RT (min) | Precursor ion (Q1) | Product ion (Q3) |
| --- | --- | --- | --- |
| [M + H]^+^ | 11.40 | 714.5 | 184.1 |
| [M + H]^+^ | 12.07 | 758.6 | 184.1 |
| [M + H]^+^ | 10.78 | 742.5 | 184.1 |
| [M + H]^+^ | 12.75 | 730.5 | 184.1 |
| [M + H]^+^ | 11.73 | 766.6 | 184.1 |
| [M + H]^+^ | 12.16 | 690.5 | 184.1 |
| [M + H]^+^ | 10.85 | 730.5 | 184.1 |
| [M + H]^+^ | 4.70 | 520.4 | 184.1 |
| [M + H]^+^ | 19.23 | 880.8 | 579.5 |
| [M + H]^+^ | 3.46 | 288.3 | 270.3 |
| [M + H]^+^ | 18.78 | 878.8 | 579.5 |
| [M + H]^+^ | 14.01 | 812.6 | 184.1 |
| [M + H]^+^ | 12.08 | 784.6 | 184.1 |
| [M + H]^+^ | 13.55 | 772.6 | 184.1 |
| [M + H]^+^ | 12.01 | 728.6 | 184.1 |
| [M + H]^+^ | 11.23 | 708.6 | 663.5 |
| [M + H]^+^ | 12.87 | 810.6 | 184.1 |
| [M + H]^+^ | 11.93 | 740.6 | 184.1 |
| [M + H]^+^ | 11.12 | 756.6 | 184.1 |
| [M + H]^+^ | 13.28 | 774.6 | 184.1 |
| [M + H]^+^ | 4.10 | 460.3 | 119.1 |
| [M + H]^+^ | 12.39 | 798.6 | 184.1 |
| [M + H]^+^ | 15.56 | 536.4 | 444.4 |
| [M + H]^+^ | 14.10 | 758.2 | 281.1 |
| [M + H]^+^ | 11.53 | 752.6 | 184.1 |
| [M + H]^+^ | 15.29 | 834.7 | 184.1 |
| [M + H]^+^ | 12.49 | 610.2 | 355.1 |
| [M + H]^+^ | 3.05 | 415.2 | 119.1 |
| [M + H]^+^ | 11.38 | 536.2 | 281.1 |
| [M + H]^+^ | 2.98 | 274.3 | 256.3 |
| [M + H]^+^ | 2.99 | 318.3 | 256.3 |
| [M + H]^+^ | 5.01 | 362.3 | 261.2 |
| [M + H]^+^ | 6.42 | 611.6 | 303.3 |

**Fig. 1 - Representative ^1^H NMR spectra and total ion chromatograms.** (A) ^1^H NMR spectra of representative plasma samples. (B) and (C) Typical metabolome total ion chromatograms of plasma samples detected by LC-(+)ESI/MS and LC-(-)ESI/MS, respectively. (D) Typical lipidome total ion chromatograms of plasma samples detected by LC-(+)ESI/MS.

**Fig. 2 - Line plots of principal component analyses of the quality control samples.** (A, B) Projection by components 1 (A) and 2 (B) of the LC-(+)ESI/MS metabolome data. (C, D) Projection by components 1 (C) and 2 (D) of the LC-(-)ESI/MS metabolome data. (E, F) Projection by components 1 (E) and 2 (F) of the LC-(+)ESI/MS lipidome data.

**Fig. 3 - Retention time deviation profiles derived from LC-(±)ESI-MS analyses.** Profiles derived from the metabolome data detected by LC-(+)ESI/MS (A). Metabolome data detected by LC-(-)ESI/MS (B). Lipidome data detected by LC-(+)ESI/MS (C).

**Fig. 4 - Score plot for PCA model of HS-free and HS groups.** (A) ^1^H NMR data. (B) LC-(+)ESI/MS metabolome data. (C) LC-(-)ESI/MS metabolome data. (D) LC-(+)ESI/MS lipidome data.

**Fig. 5 - Validation plots of the partial least squares discriminant analysis models acquired through 999 permutation tests.** (A) NMR data for HS-free and HS groups. (B) LC-(+)ESI/MS metabolome data for HS-free and HS groups. (C) LC-(-)ESI/MS metabolome data for HS-free and HS groups. (D) LC-(+)ESI/MS lipidome data for HS-free and HS groups.

**Fig. 6 -** **Differentiation of HS-free and HS groups using multivariate analysis.** OPLS-DA plots of NMR data (A). OPLS-DA plots of LC-(+)ESI/MS data for the plasma metabolomes (B). OPLS-DA plots of LC-(-)ESI/MS data for the plasma metabolomes (C). OPLS-DA plots of LC-(+)ESI/MS data for the plasma lipidomes (D).

**Fig. 7 - S-plot of OPLS-DA model.** (A) NMR data for HS-free and HS groups. (B) LC-(+)ESI/MS metabolome data for HS-free and HS groups. (C) LC-(-)ESI/MS metabolome data for HS-free and HS groups. (D) LC-(+)ESI/MS lipidome data for HS-free and HS groups.

**Fig. 8 - Loading plot with jack-knifed confidence intervals.** (A) NMR data for HS-free and HS groups. (B) LC-(+)ESI/MS metabolome data for HS-free and HS groups. (C) LC-(-)ESI/MS metabolome data for HS-free and HS groups. (D) LC-(+)ESI/MS lipidome data for HS-free and HS groups.

**Fig. 9 - Raw data plots of peak areas of glucose ^1^H signal in 5.245 ppm (A) and acetate ^1^H signal in 1.925 ppm (B).** The peak areas of glucose showed no overlapping between groups; however, the acetate overlapped across two groups and should be deleted.

**Fig. 10 - Visualization of the discriminatory powers of individual and combined diagnostic biomarkers.** **Heat maps showing the discriminative capacities of selected biomarkers (A). The red and blue colors represent high and low area under the curve (AUC), respectively. Receiver operating characteristic (ROC) curves were generated per metabolite (B).**
